# Nontoxic Fluorescent Nanoprobes for Multiplexed Detection and 3D Imaging of Tumor Markers in Breast Cancer

**DOI:** 10.3390/pharmaceutics15030946

**Published:** 2023-03-15

**Authors:** Pavel Sokolov, Galina Nifontova, Pavel Samokhvalov, Alexander Karaulov, Alyona Sukhanova, Igor Nabiev

**Affiliations:** 1 Laboratory of Nano-Bioengineering, National Research Nuclear University MEPhI (Moscow Engineering Physics Institute), 115522 Moscow, Russia; 2 Laboratoire de Recherche en Nanosciences, LRN-EA4682, Université de Reims Champagne-Ardenne, 51100 Reims, France; 3 Department of Clinical Immunology and Allergology, Institute of Molecular Medicine, Sechenov First Moscow State Medical University (Sechenov University), 119146 Moscow, Russia

**Keywords:** breast cancer, biomarkers, tumor microenvironment, optical imaging, 3D imaging, fluorescent nanocrystals, single domain antibodies, quantum dots, conjugation

## Abstract

Multiplexed fluorescent immunohistochemical analysis of breast cancer (BC) markers and high-resolution 3D immunofluorescence imaging of the tumor and its microenvironment not only facilitate making the disease prognosis and selecting effective anticancer therapy (including photodynamic therapy), but also provides information on signaling and metabolic mechanisms of carcinogenesis and helps in the search for new therapeutic targets and drugs. The characteristics of imaging nanoprobe efficiency, such as sensitivity, target affinity, depth of tissue penetration, and photostability, are determined by the properties of their components, fluorophores and capture molecules, and by the method of their conjugation. Regarding individual nanoprobe components, fluorescent nanocrystals (NCs) are widely used for optical imaging in vitro and in vivo, and single-domain antibodies (sdAbs) are well established as highly specific capture molecules in diagnostic and therapeutic applications. Moreover, the technologies of obtaining functionally active sdAb–NC conjugates with the highest possible avidity, with all sdAb molecules bound to the NC in a strictly oriented manner, provide 3D-imaging nanoprobes with strong comparative advantages. This review is aimed at highlighting the importance of an integrated approach to BC diagnosis, including the detection of biomarkers of the tumor and its microenvironment, as well as the need for their quantitative profiling and imaging of their mutual location, using advanced approaches to 3D detection in thick tissue sections. The existing approaches to 3D imaging of tumors and their microenvironment using fluorescent NCs are described, and the main comparative advantages and disadvantages of nontoxic fluorescent sdAb–NC conjugates as nanoprobes for multiplexed detection and 3D imaging of BC markers are discussed.

## 1. Introduction

Breast cancer (BC) is one of the most common cancers worldwide and ranks fifth in mortality [1]. As with other cancers, the effectiveness of BC treatment depends on early diagnosis. If the disease is already progressing, then selection of effective therapy requires data on the gene expression profile, mutations and polymorphism, as well as the secretome and proteome of tumor cells and their microenvironment [2,3]. However, the mutation rate in BC is lower than in other common cancers, e.g., it is about ten times lower than in lung cancer, stomach cancer, and melanoma. This makes sequencing methods less relevant to the diagnosis of this disease [4,5]. Secretome and proteome studies are still very expensive, cumbersome, and, hence, unlikely to be used in clinical practice in the near future. Therefore, BC diagnosis mainly relies on immunological methods and focuses on the quantitative profiling and spatial distribution of both cancer markers and other biomarkers associated with BC, including tumor microenvironment ones. The active development of cancer immunotherapy methods [6,7] makes the study of the tumor microenvironment an increasingly important scientific and clinical task. The most common diagnostic approach in this area is to detect typical cancer markers by means of immunohistochemical (IHC) examination of biopsy and/or postoperative specimens, the IHC detection of the markers being considered a 100% confirmation of the diagnosis [8]. However, IHC methods are usually limited to hematoxylin and eosin staining and staining of a few BC-specific biomarkers, which is often insufficient for prescribing adequate therapy, monitoring its effectiveness, or identifying new targets for immunotherapy. Advanced minimally invasive biopsy methods, such as punch biopsy, use a needle about 1 mm in diameter to collect the biomaterial without disrupting the tumor structure or its blood vessels. This makes it possible to examine several tumor parts, which is particularly important because of the high heterogeneity of tumor structures [9]. An undoubted advantage of methods based on IHC staining is that they are easy to bring into clinical practice, because the testing procedures are routine and the equipment is readily available. The average thickness of sections for IHC tests is about 5 μm, but this is not enough for establishing the mutual arrangement of tumor biomarkers and restoring the bulk structure of the tumor and its microenvironment. McCambell et al. [10] have shown that the thickness of sections for IHC studies significantly affects the efficiency of marker staining and should be selected according to the recommendations of antibody (Ab) manufacturers, based on the analysis of a large number of tumor specimens.

**Figure 1:**
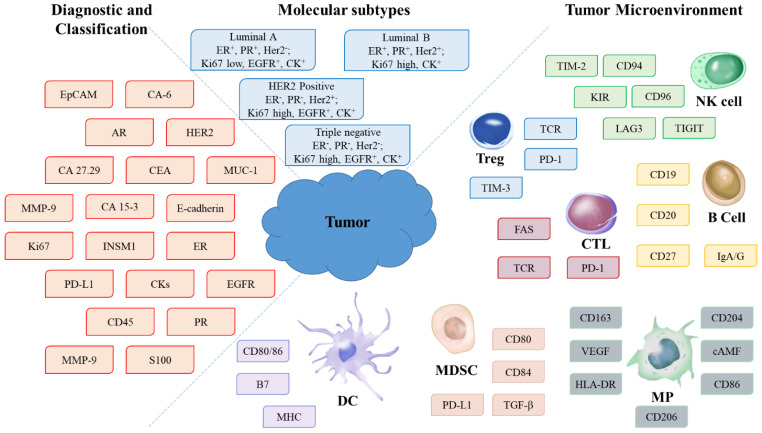
Biomarkers of the tumor and tumor microenvironment for diagnosis and classification of breast cancer and selection of the treatment. Designations: Treg, regulatory T cells; NK, natural killer cells; CTL, cytotoxic T lymphocytes; DC, dendritic cells; MDSC, myeloid-derived suppressor cells; MP, macrophages.

A promising alternative to IHC studies is multiplexed three-dimensional (3D) detection of biomarkers and monitoring of their profiles in BC biopsy specimens. The systems for this analysis combine fluorescent tags, biological capture molecules, procedures for obtaining thick tissue specimens, and 3D imaging systems, which provide comprehensive data that are sufficient to precisely classify BC, select effective therapy, assess the risk of metastasizing, and predict the treatment outcome. This review is aimed at highlighting the importance of an integrated approach to diagnosis, including the detection of tumor markers and tumor microenvironment biomarkers, as well as the need for their quantitative profiling and imaging of their mutual location (Figure 1). We will also review the most advanced approaches to 3D detection in thick tissue sections and fluorescence imaging using ultrasmall fluorescent probes based on inorganic fluorescent nanocrystals and single-domain antibodies (sdAbs). In conclusion, we will analyze the advantages of ultrasmall fluorescent nanoprobes and prospects of their use for 3D imaging.

## 2. Tumor Biomarkers in the Diagnosis, Classification, and Treatment of Breast Cancer

The current approach to cancer diagnosis is mainly aimed at integrated detection of biomarkers rather than identification of individual biomarkers, in order to precisely determine the tumor types and tissues from which they originate, differentiate between primary tumors and metastases, and provide additional information that can help make a correct prognosis, select the most effective treatment option, and monitor the treatment efficacy. There are several approaches to BC classification, based on histologic groups, molecular subtypes, grade of histologic malignancy, TNM (tumor, nodes, metastasis) staging, and DNA and RNA data. We will consider the former two classifications, which are based on the detection of protein biomarkers by IHC methods. The biomarkers are used to differentiate between benign neoplasms and malignant tumors, including invasive and in situ forms, because the data on tissue structure obtained using hematoxylin and eosin staining are often insufficient.

### 2.1. Markers Differentiating between Benign Neoplasms and Invasive or In Situ Carcinomas

Precise differentiation between benign tumors, borderline neoplasms, and carcinomas in situ is necessary because only the latter two forms should be completely excised, whereas benign tumors only require regular follow-up. These forms can be differentiated by IHC methods with hematoxylin and eosin staining, because normal hyperplasia is characterized by heterogeneous cells, fuzzy margins, and variable shape and size, whereas atypical hyperplasia and ductal carcinoma in situ are represented by populations of identical cells with clear cell boundaries around empty areas. Markers that are used to differentiate invasive and in situ cancers from morphologically similar benign neoplasms are expressed by myoepithelial cells. For example, benign neoplasms, except for microglandular adenosis, retain the myoepithelial cell layer, whereas invasive malignant tumors developing from epithelial tissue cells lose it [11]. For example, luminal cells express the cytokeratins (CKs) CK7, CK8, CK18, and CK19; basal cells express CK5, CK6, CK14, and CK17; and myoepithelial cells, CK5, CK14, and CK17. Otterbach et al. [12] analyzed 699 breast lesions and showed that CK5 and CK6 could be used for differentiation. These two markers were highly expressed in 87.6% of benign tumors, whereas no expression was found in 47.4% cases of atypical ductal hyperplasia and in 96.3% cases of ductal carcinoma in situ. Duivenvoorden et al. [13] described the use of myoepithelial markers, including smooth muscle actin (SMA), smooth muscle myosin heavy chain (SMMHC), CK14, and p63, for differentiating mammary intraepithelial neoplasia from invasive carcinoma in mouse models with transgenic tumors. Their results, summarized in Table 1, indicate that only integrated detection of several biomarkers allows correct identification of the tumor type. Ki-67, SMMHC, and CK14 are the most informative biomarkers, but even their detection is sometimes insufficient for correct differentiation between the early stages of preinvasive and invasive lesions. Kesse-Adu and Shousha [14] studied 117 samples of tumor tissue from women with a confirmed diagnosis of invasive breast carcinoma and found that myoepithelial markers were expressed in one-third of the cases, which also indicates the need for an integrated approach to BC diagnosis.

Martinez et al. [15] stained 65 specimens using Abs against the estrogen receptor (ER), CK5, progesterone receptor (PR), and Bcl-2 apoptosis regulator to test which biomarkers or their combinations were the best for discriminating between benign and malignant neoplasms. Staining with Abs against Bcl-2 yielded no statistically significant results. Staining for PR showed a diffuse pattern in 48% of atypical ductal hyperplasia specimens, 60% of ductal carcinoma in situ specimens, and 13% of common ductal hyperplasia specimens, which does not allow these markers to be used for diagnosis. Staining with Abs against ER resulted in diffuse staining in 94% of specimens from patients with confirmed atypical ductal hyperplasia and ductal carcinoma in situ, whereas none of the 32 specimens from patients with usual ductal hyperplasia was stained. Similarly, CK5 staining exhibited a very high potential for tumor discrimination: 96% of specimens from patients with confirmed atypical ductal hyperplasia and ductal carcinoma in situ were not stained or had only isolated stained foci, whereas all specimens with usual ductal hyperplasia were diffusely stained. The combined use of both markers, ER and CK5, increases the accuracy of discrimination between benign and malignant neoplasms to 97%.

Papillary lesions of the mammary gland are characterized by a fibrous stroma with blood vessels, which support the proliferation of epithelium. This heterogeneous group includes benign intraductal papilloma, atypical papilloma, papillary ductal carcinoma in situ, and intracystic and solid papillary carcinomas. Recognition of papillary structures is a routine task, but their subclassification is often quite challenging. This is usually done by specific staining of various high molecular weight CKs (e.g., CK5, CK6, and CK14), myoepithelial markers (e.g., CD10, S100 protein, and calponin), neuroendocrine markers (e.g., chromogranin and synaptophysin), and hormone receptors (e.g., ER and PR) [16,17]. However, it is difficult to distinguish benign intraductal papilloma from atypical papilloma using only hematoxylin and eosin staining, nor does staining for CK5/6 and ER, which is the most commonly used for differentiating between these two tumors, always yield unambiguous results [18,19]. In addition, the epithelium of benign intraductal papilloma may exhibit both common ductal hyperplasia and apocrine metaplasia, in which CK5/6 is not expressed at all [19]. At the same time, different forms of neoplasia can sometimes be identified by comparing the sizes of the affected areas. Wen and Cheng [20] analyzed 34 studies in which the diagnosis was initially made using punch biopsy, with a sample taken with a thin needle, and then the results were verified by IHC staining of surgically removed material. As a result, 346 cases initially classified as nonmalignant lesions were subsequently reclassified as malignant ones; the main reason for this significant underestimation of malignancy being atypical papillary lesions, which were insufficiently examined in small tissue sections.

### 2.2. Markers Differentiating between In Situ Carcinomas: Lobular and Ductal Carcinomas

E-cadherin [21] and CK8 [22] are biomarkers used to differentiate between lobular and ductal carcinomas. Singhai et al. [23] analyzed 276 tissue samples from BC patients who had undergone radical mastectomy. The results showed that the loss of E-cadherin expression accompanied the lobular form in 98% of cases, and its expression accompanied the ductal form in 88% of cases, so that testing for this biomarker alone could not provide a sufficiently reliable answer. Lehr et al. [24] analyzed 48 samples and found that all the 33 ductal carcinoma specimens were stained with Abs against E-cadherin, whereas 15% of lobular carcinoma specimens remained unstained. To improve the accuracy of differentiation, these authors suggested additional staining with anti-CK8 Abs. In the ductal form, peripheral staining revealing clusters of tumor cells was prevailing, whereas in the lobular form, a circular perinuclear distribution within individual tumor cells was the predominant pattern.

### 2.3. Markers of Invasive Carcinomas: Lobular and Ductal Carcinomas

Invasive carcinomas are the most common BC forms. Ductal carcinoma is the most frequently diagnosed form, accounting for about 55% of all BC cases [25], and invasive lobular carcinoma accounts for up to 15% of all cases [26]. Among invasive breast carcinomas, the so-called nonspecific invasive ductal carcinoma accounts for as many as 75% of all tumors, and precisely this form is usually meant when the term BC is used [27]. Nonspecific invasive ductal carcinoma is characterized by a wide spectrum of morphological variations and clinical manifestations, a heterogeneous growth type, and a varying degree of ductal differentiation, ranging from more than 70% to a complete absence. Therefore, it is almost impossible to select specific markers for them, and they are divided into molecular subtypes [28], which we will discuss in more detail below.

Metaplastic carcinoma is one of the most malignant ductal carcinomas. As a rule, it is an invasive tumor, because its elongated spindle-shaped cells can form short or long bundles of complex shapes [29]. Several biomarkers are used simultaneously to diagnose this type of tumors, because staining with only one may give an incorrect result due to heterogeneity of the tumor tissue. Rakha et al. [30] analyzed immunoprofiling data on 730 cases of metaplastic breast carcinoma described in different research articles and concluded that the basal CKs (CK5, CK6, and CK14) were found in 70% of cases, while the luminal CKs (CK8, CK18, and CK19) were found in 30–60% of cases. Myoepithelial markers, mainly p63, were also quite common, whereas ER, PR, and human epidermal growth factor receptor 2 (HER2) were usually absent, and CD34, typically found in phylloid tumors, was always absent. Koker et al. [31] also confirmed that positive staining for p63 occurred in 86.7% of cases, although this marker was also found in infiltrating phylloid tumors. As a rule, metaplastic carcinoma exhibits positive staining for the S100, epidermal growth factor receptor (EGFR), SMA, CD10, and vimentin markers [32] and is negative for ER, PR, HER2, BCL2, and CD34 [33,34].

Apocrine carcinoma is also a readily well differentiable BC, its markers being HER2, EGFR, and androgen receptor (AR) [35], but it can easily be confused with pleomorphic lobular carcinoma. Pleomorphic lobular carcinoma may be positive for AR and HER2 [36], but it usually loses the expression of E-cadherine [37], in contrast to apocrine carcinoma, which retains the expression of this marker [38].

### 2.4. Markers for Determining whether the Breast Tunor Is Primary or Metastatic

The above examples show that simultaneous detection of multiple markers is necessary for correct differentiation of BC types and subsequent selection of adequate treatment. Another factor important for selecting the right treatment is determining the place of origin of the tumor, i.e., whether the tumor is primary or it is a metastasis of a tumor located elsewhere. This question is also answered by means of IHC tests, which, as with the identification of the tumor type, requires simultaneous detection of different biomarkers. The most prognostically reliable markers of metastases to the mammary gland are napsin A and TTF-1 for lung cancer [39,40]; WT1, PAX-8, and CA125 for ovarian cancer [41]; CK20 and CDX2 for stomach cancer [42,43]; synaptophysin and chromogranin A for neuroendocrine tumors [44]; and HMB-45, S100, and Melan A for melanoma [45,46,47]. At the same time, metastatic breast lesion is quite a rare event, accounting for only 2% of all BC cases. Nevertheless, detection of these markers can significantly accelerate diagnosis and selection of effective therapies.

### 2.5. Markers for Determining the Prognosis and Selecting the Treatment

More than fifty biomolecules serve as BC diagnostic markers, allowing the detection and correct classification of the tumor and estimation of its invasiveness. The pool of prognostic biomarkers, which are used to predict the most probable clinical course, the probability of recurrence, and the biological aggressiveness of the tumor, is no smaller.

The ER and PR markers mentioned above are also prognostic markers. ER- and PR-positive tumors are characterized by lower growth rates, longer remission periods, and a higher survival of patients [48]. ER and PR are used to predict the response to endocrine (hormonal) therapy and allow its efficacy to be determined at the early stages of tumor growth. In this case, if at least 1% of cells express the receptors, the tumor is considered to be positive for these markers. Patients whose tumors express ER respond well to endocrine therapy, whereas it is ineffective for patients with ER-negative tumors [49]. On the other hand, tumors that do not express the hormone receptors respond better to chemotherapy than do the tumors expressing them [50].

HER2 is not only a prognostic biomarker, but also one of the few cancer markers approved for clinical and laboratory studies. Despite the wide choice of diagnostic markers, the U.S. Food and Drug Administration (FDA) has approved only four markers for BC diagnosis as of 2021, including the cancer antigens CA 15-3, CA 27-29, HER2, and circulating tumor cells (in case body fluids are analyzed) [51]. A high expression of HER2, which indicates activity of several tumor growth pathways, occurs in 15–30% of BC cases [52]. Prognosis of these tumors is always unfavorable, as they often metastasize to lymph nodes. However, patients with these tumors respond well to treatments targeting this receptor, such as trastuzumab- or anthracycline-based therapies [53,54].

Biomarkers of the cell cycle, such as Ki-67, p21, and p27, as well as cyclins D and E, are traditionally used to assess the proliferative potential of tumor cells [55]. Ki-67 is one of the most frequently used biomarkers of proliferative activity, as a rule, in conjunction with other biomarkers. For example, patients with ER-positive, HER2-negative tumors and a Ki-67 level below 5% need no chemotherapy, as do for those with ER-positive, HER2-positive tumors and a Ki-67 level below 30% [56].

There are many approaches to classifying BC into molecular subtypes, based on gene expression and proteomic profiles, but the most widely used approach is IHC detection of three (ER, PR, and HER2) to six or more (ER, PR, HER2, Ki-67, EGFR, CKs, and some others) biomarkers [57,58]. As a rule, four subtypes are distinguished: basal (10–15% of cases), luminal A (50–60%), luminal B (15–20%), and HER2-enhanced (15–20%) subtypes. The basal subtype, also called triple-negative subtype (negative for the ER, PR, and HER2 markers) is also characterized by a high Ki-67 level and the presence of EGFR and CKs. This subtype has an unfavorable prognosis with a remission shorter than five years. It is resistant to endocrine and HER2-targeted therapies. In addition, it is more often accompanied by an enhanced expression of programmed cell death ligand 1 (PD-L1) than other subtypes, which gives hope for the development of effective drugs targeting it [59]. The luminal A subtype is characterized by an ER-positive, HER2-negative phenotype with a PR level higher than 20% and a low Ki-67 level. The expressions of EGFR and CKs are usually weak or absent. The prognosis for survival is good, and the remission, depending on additional factors, may be longer than ten years [60]. Most tumors of this subtype respond well to endocrine therapy and, in some cases, to chemotherapy. The luminal B subtype is characterized by higher Ki-67 and HER2 expression rates and a lower PR expression rate (<20%) compared to the luminal A subtype. The prognosis is worse than that of the luminal A subtype. This subtype is less susceptible to endocrine therapy but more susceptible to chemotherapy, with a remission shorter than ten years [61]. In the HER2-enhanced subtype, ER and PR are not expressed, and the HER2 expression rate is high. The Ki-67 level is typically high, and EGFR and CKs are detected more frequently than in the preceding two subtypes [58]. The prognosis is worse compared to the luminal A and B subtypes but slightly better compared to the basal subtype, with a remission shorter than ten years. This subtype responds to HER2-targeted therapy and anthracycline chemotherapy [62].

It is worth noting that lifestyle, particularly physical activity and diet, may play an important role in the development and treatment of BC. Chen et al. [63] analyzed 86 research papers that addressed the impact of obesity on BC development, treatment/prevention approaches, and posttreatment pathologies. It was shown that obesity is associated with activation of multiple oncogenic pathways, including leptin signaling network and oxidative stress mechanisms; moreover, obesity is a trigger of inflammation and oncogenesis through accumulation of activated M1 macrophages in adipose tissue. Moderately overweight patients have shown better survival outcomes after extensive chemotherapy treatments, but at the same time, obesity is associated with higher morbidity and mortality rates for BC.

Cancer cells need a lot of energy to maintain its growth, so research on the mitochondria of cancer cells is an important scientific and practical task in terms of cancer diagnosis and therapy. Indeed, by manipulating oxidative phosphorylation and glycolysis mitochondria genes the tumor growth and metastasis process can be restrained. Mutations in mitochondrial DNA are commonly found in cancer cells and promote the rewiring of bioenergetics and biosynthesis of various metabolites, including oncometabolites. For more details, you can read the overview about role of mitochondria in cancer metabolism written by Liu et al. [64]. Chen et al. summarized more detailed information on mutations and epigenetic regulation of mitochondrial DNA and its impact on mismanagement of reactive oxygen species cascade and its association with cancer prognosis as well as usage of mutations and epigenetic modifications of mitochondrial DNA as viable markers for early diagnosis and targeted therapy of BC [65].

Actively dividing cells of solid malignant tumors are known to substantially change their environment, because they need oxygen and nutrients for growth. As a result, they stimulate the formation of stroma-like structures. The formation of these structures, together with the extracellular matrix and other components, may strongly affect the efficacy of therapy by changing the efficiency of drug delivery and suppressing the body’s immune response. All this is due to the formation of a complex 3D tumor microenvironment, with heterogeneous distribution of cells, lymphocytic infiltration, and other characteristic features. This, together with the development of immunological methods of cancer treatment, has raised researchers’ and clinicians’ interest in developing approaches to the study of tumor microenvironment, detection of relevant biomarkers, and analysis of its 3D structure. This is expected to make the development of new drugs more efficient and their prescription more correct. It should be noted that, despite the development of molecular, transcriptomic, genetic, secretomic, and proteomic methods of analysis, IHC studies are still necessary because they provide information on the mutual spatial arrangement of individual biomarkers, which is required for morphological analysis, precise determination of the tumor type, prognosis, and selection of the optimal therapy. However, the available data on more than fifty diagnostic and prognostic biomarkers and potential of their simultaneous detection cannot be implemented by classical IHC methods. Therefore, new methodologies for multiplexed imaging of tumor biomarkers and its microenvironment are being developed.

## 3. Tumor Microenvironment Biomarkers in the Diagnosis, Classification, and Treatment of Breast Cancer

Many researchers compare a tumor and its microenvironment to a separate organ with its own blood supply, lymphatic and nervous systems, its own immune cells, and an extracellular matrix containing growth factors, hormones, and other components that ensure tumor development and its evasion of therapy. The tumor microenvironment contains exosomes of cancer and normal cells and regulatory RNAs that alter the integrated characteristics of tumor tissues. Abnormal hypoxic conditions, concentrations of various components atypical of normal tissues, and metabolic abnormalities affect the efficiency of a selected treatment strategy. Therefore, affecting cancer cells alone is often insufficient for efficacious therapy, and an integrated approach addressing the changes in signaling pathways, immune response, and many other natural body defense mechanisms is required [66]. In addition, the biomarkers of tumor microenvironment components that enter the bloodstream can be used to estimate the clinical picture of BC progression [67], as well as to determine the likely effective treatments [68].

PD-1/PD-L1 is one of the immune checkpoint biomarkers and when PD-L1 binds to PD-1 through a signaling cascade, there is a decrease in proliferation of antigen-specific T lymphocytes in the lymph nodes and a decrease in apoptosis in regulatory T lymphocytes, which ultimately leads to immune system suppression. This ability to suppress the immune system is found in various types of cancer, as the tumor and its microenvironment actively produce PD-L1. Zhang et al. [69] reviewed more than 2500 cases of BC where the PD-L1 expression level was detected to find that its increased expression is associated with a high probability of lymph node metastases and tumor negativity for either ER alone or for three markers, ER, PR, and HER2, at once. It should be noted that PD-L1 is detected in lymphocytes infiltrating the tumor, which are the dominant type of ER-negative T cells, rather than in cancer cells themselves [70]. The regulatory mechanisms of PD-L1 expression are complex and diverse and include both various processes in cancer cells and interactions between tumor and immune cells, such as the WNT signaling pathway [71] and suppression of the PD-L1 expression when ER is inhibited [72].

Immunotherapies that block immune checkpoints, such as PD-1/PD-L1 or CTLA-4, thereby reactivating T cell immune responses to tumor cells and disrupting tumor immunosuppression, are effective in many cases, but some patients do not respond to these treatments. An alternative is to target the LAG-3 protein, also called CD233. Liu et al. [73] analyzed clinical information on about 3000 biopsy specimens from BC patients to show that LAG-3 is expressed by immune cells, including T, B, and dendritic cells, located in the tumor microenvironment. LAG-3 downregulates T cell activation, enhances differentiation of T regulatory cells, and decelerates the proliferation of natural killer cells, which leads to the conclusion that expression of this biomarker is associated with unfavorable clinical prognosis. Combined immunotherapy targeting LAG-3 and PD-1/PD-L1 has been shown to be effective, but its clinical trials are still underway [74] because of a high risk of side effects, as was the case with combined therapy targeting CTLA-4 and PD-1/PD-L1 [75].

Zhang et al. [76] studied another potential target for cancer immunotherapy, T cell immunoreceptor with Ig and ITIM domains (TIGIT), in order to establish the correlation between the expression rate of this biomarker and the observed clinical picture of BC. Data on several thousand patients showed that TIGIT could alter the immune response to tumor cells by affecting not only T cells, but also other cells of the immune system. TIGIT can be expressed simultaneously with PD-1 on the surface of tumor-infiltrating T cells. Typically, intense TIGIT expression indicate a highly aggressive tumor and an unfavorable clinical prognosis. Stamm et al. [77] used in vitro models to show that blockage of TIGIT expression led to immune-cell-associated lysis of SKBR-3, BT549, and other tumor cells. This makes TIGIT a potentially important target for hormonal BC therapy. For example, combined therapy with tiragolumab (an antibody against TIGIT) and atezolizumab (an antibody against PD-L1) causes a synergistic effect and enhances the antitumor response of the immune system [78].

**Figure 2:**
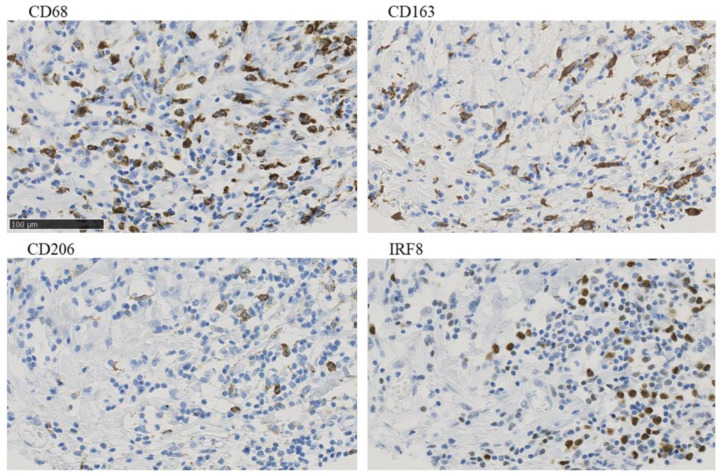
Example of CD68, CD163, CD206, and IRF8 immunostaining in serial sections of a single tumor from a patient, with × 40 magnification of a selected area. Adapted from Bobrie, A. et al. [80].

Macrophages constitute another component of the tumor microenvironment that can promote tumor development under certain conditions. For example, clusters of macrophages that actively express the macrophage mannose receptor (MMR) and have a proangiogenic effect are found in hypoxic regions of tumors. Data reported by Debacker et al. [79] confirm these conclusions. They analyzed 27 clinical cases and found that patients with high levels of MMR expression in tumor microenvironment macrophages exhibited a significantly lower survival rate than patients without overexpression of this biomarker. The macrophage population infiltrating the tumor is very heterogeneous, as the phenotype and function of macrophages can vary depending on environmental conditions. Bobrie et al. [80] analyzed tumors of basal type of BC in 285 patients and concluded that it is necessary to determine up to four markers of macrophages at once for their more accurate differentiation (CD68, IRF8, CD163, CD206) (Figure 2). It is also worth noting that the authors performed staining of four consecutive sections rather than multiplex staining, which would allow the coexpression of these markers to be determined.

Signal-regulatory protein α (SIRPα) is expressed on the surface of neurons, myeloid cells, and hematopoietic stem cells, including macrophages, neutrophils, and dendritic cells. Its interaction with CD47 on the surface of tumor cells forms a signaling pathway that allows tumor cells to avoid phagocytosis by macrophages. The results of many research papers and phase I/II clinical trials confirm that CD47 inhibition leads to stimulation of tumor cell phagocytosis by macrophages. However, typical side effects of this treatment are anemia and other disorders, which requires additional study of this complex signaling pathway [81].

**Figure 3:**
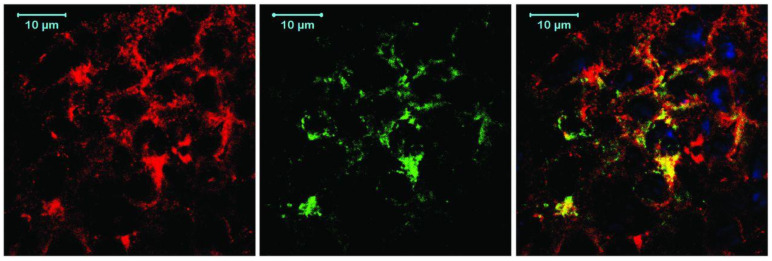
Immunofluorescence staining for CRIg and CD68 in synovium of inflamed knee (*n* = 3). Formalin-preserved cryosections of inflamed knee synovium were stained with anti-CD68-biotin and Alexafluor568-streptavidin (**left**) and with hemaglutinin-tagged NbV4m119 and Alexafluor488-conjugated antihemaglutinin antibody (**middle**). Merged image (**right**) shows colocalization of CRIg expression with subset of CD68-positive macrophages. 4′,6-diamidino-2-phenylindole (DAPI) (blue) was used as nuclear stain. Reproduced from Zheng et al. [83].

V-set and immunoglobulin domain-containing protein 4 (VSIG4) is also sometimes expressed on the surface of macrophages. It may promote proliferation, invasion, and migration of BC cells [82]. Zheng et al. [83] have developed the NbV4m119 sdAb against VSIG4 and proved its specificity in experiments on simultaneous fluorescent staining of macrophages in the area of inflammation (Figure 3). One of the possible mechanisms of VSIG4 carcinogenicity is suppression of T cell proliferation and cytokine synthesis; therefore, inhibition of its expression can be another line of hormonal therapy for tumors [84].

Ni et al. [85] analyzed 13 studies involving a total of more than 5000 patients to find a correlation between the expression of CD68 and CD163 on the surface of tumor-infiltrating macrophages and the clinical signs of BC. Many of CD68- and CD163-positive BC cases were accompanied by lymph node metastases, high levels of Ki-67 expression, or low levels of ER and PR expressions. Macrophages positive for CD163 alone were frequently associated with poor survival. Shabo et al. [86] showed that CD163^+^ macrophages were the most likely to form through the fusion of macrophages with cancer cells, because this could not be explained by simple intercellular interaction. In addition, CD163^+^ macrophages in the tumor microenvironment are associated with an increased risk of tumor recurrence after radiotherapy, which makes this biomarker prognostically important [87].

T cells constitute an element of the natural defense of the body. The presence of CD8-positive T cells in the tumor microenvironment indicates a better clinical prognosis of BC in young women and, conversely, a worse prognosis in older age groups [88]. In the case of basal-type BC, tumors infiltrated by T cells with a high level of CD8 expression are usually characterized by higher concentrations of interferons alpha and gamma (IFN-α and IFN-γ) and immune cells, as well as a high expression level of immune checkpoint molecules, which ultimately results in a better prognosis for survival. The level of this biomarker also reflects the efficacy of hormonal therapy, which makes it an important prognostic marker [89]. So et al. [90] have shown that a large number of CD8^+^ T cells in tumors recurring after surgery is a marker of the success of immunotherapy.

CD69 is expressed on the surface of T and B cells and is an early marker of lymphocyte proliferation. For example, expression of CD69 on activated T cells is essential for the localization and accumulation of these T cells in inflamed tissues. Mita et al. [91] studied how the expression of this biomarker affected T cells infiltrating the tumor and tumor development in general. Experiments on CD69-positive and negative BALB/c mice with 4T1-luc2 murine BC cells inoculated into the mammary fat layer showed that the tumors grew slower and produced fewer lung metastases in CD69^–^ mice than in CD69^+^ mice. CD69^–^ mice were also characterized by a greater amount of CD8^+^ lymphocytes with an enhanced synthesis of cytokines, including IFN-α and IFN-γ. This indicates that CD69 is responsible not only for T cell accumulation, but also for their effector antitumor properties. Moreover, administration of anti-CD69 antibodies to CD69^+^ mice significantly reduced the tumor volume and increased its infiltration by CD4^+^ and CD8^+^ T cells, which confirms the efficacy of immunotherapy targeting CD69.

In conclusion, it should be noted that planning of adequate radio-, chemo- and immunotherapies is impossible without comprehensive information on multiple biomarkers. However, it is not enough to know only the expression rates of these biomarkers, because the tumor microenvironment is highly heterogeneous, and local structural characteristics provide key information for selecting the right therapy and predicting the outcome. Below, we will focus on how biomarkers are detected and which affinity molecules and labels can be used for simultaneous multiplexed detection. Special emphasis will be made on the penetrability of these labels into thick tissue sections, because this is necessary for determining the complex 3D structure of the tumor and for spatially mapping the biomarkers.

## 4. Studying the 2D/3D Structure of the Tumor and Its Microenvironment

**Figure 4:**
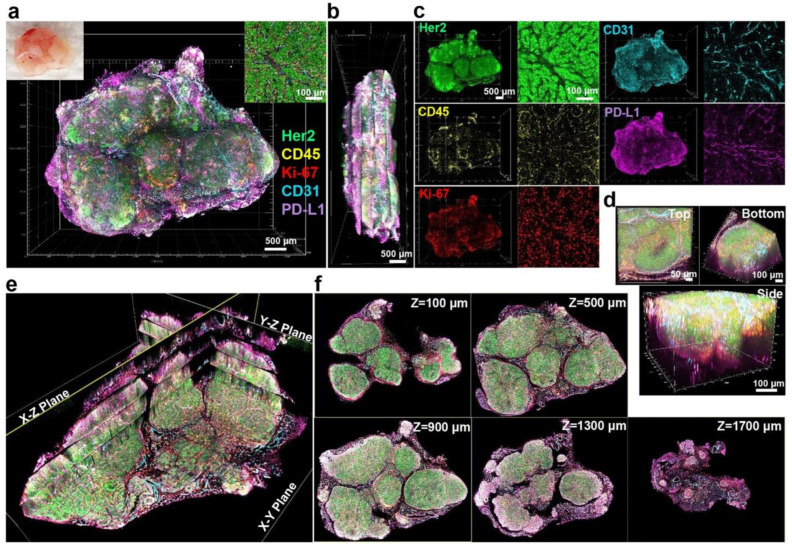
3D mapping of multiple tumor microenvironment components and biomarkers in a whole mouse mammary tumor. (**a**) 3D rendering of reconstructed tumor mass excised from the mammary gland of a BALB-NeuT mouse obtained by fusing images of five 400 μm macrosections each immunostained for Her2 (green), CD45 (yellow), Ki-67 (red), CD31 (cyan), and PD-L1 (magenta). Scale bar: 500 μm. Left top insert shows tumor tissue prior to macrosectioning. Right top insert displays representative 2D image of immunostaining. Scale bar: 100 μm. (**b**) Lateral view of the reconstructed tumor. Scale bar: 500 μm. (**c**) 3D (left) and 2D (right) channel images for each marker, showing distinct patterns of expression of the cellular markers and biomarkers across tumor. Scale bars: 500 μm (left) and 100 μm (right). (**d**) Distinct views of a volume within the rendered tumor model (580 × 580 × 400 μm). Scale: 50 μm (left) and 100 μm (right, bottom). (**e**) Tomographic visualization of the reconstructed tumor image with multiple orthogonal planes (X-Y, X-Z, Y-Z planes). (**f**) Serial tomographic sections of X-Y planes at different Z-stack depths (100, 500, 900, 1300, and 1700 μm). Reproduced from Lee et al. [94].

As shown above, information on the integrated expression profile of various biomarkers and their structural distribution is usually required for correctly predicting the efficacy of BC immunotherapy. This is of special importance because the tumor microenvironment has a complex structural organization, with heterogeneous cell distribution and local features, such as hypoxic areas, or, conversely, a high concentration of blood vessels, all of which may affect the efficacy of therapy. Traditionally, thin sections of fixed tumor tissue are made, and chromogenic staining is performed using specific labeled Abs to detect individual biomarkers. In addition, hematoxylin staining is used to image the cell nucleus and sites of nucleic acid accumulation, while eosin staining is used to image the cytoplasm and mass protein accumulations. However, the chromogenic IHC study is almost entirely unsuitable for either multiplexed analysis (and hence, integrated biomarker assessment) or determination of the 3D structure of the tumor, as it is suitable for studying only thin sections. Although 3D reconstruction of a tumor from multiple consecutive thin sections yields good results [92], it is almost never used in clinical practice, because it is time consuming and technically demanding. It should be noted that multiplexed IHC analysis with colorimetric staining is also possible. For example, Koh et al. [93] have developed a multiplexed IHC assay for detecting ten biomarkers of a tumor and its microenvironment that includes ten sequential stages of demasking of the antigen, blockage of nonspecific binding, treatment of the specimen with detection antibodies, staining, scanning, and removal of primary detection antibodies. Obviously, this sequential staining takes a long time. Moreover, the repetitive procedures of demasking, staining, and antibody removal are likely to damage the antigen molecules and, hence, affect the validity of the results. Lee et al. [94] described the multiplexed examination of the tumor microenvironment using a modified protocol for imaging whole tumors or individual organs. In the original method described by Chung et al. [95], optical transparency is achieved through a long process of infusion of formaldehyde and hydrogel monomers into the organ (for one to three days), hydrogel polymerization in the tissue (for about three days), and subsequent electrophoretic purification with an ionic detergent (for five to nine days) to wash off lipids from the specimen while leaving proteins bound to the hydrogel. The obvious disadvantage of this method is the enormous time consumption, which makes it unsuitable in clinical use, where quick and simple methods are required. In addition, this procedure often leads to tissue deformation, biomarker loss, and uneven immunostaining [96]. Lee et al. [94] optimized this method as follows. To prepare sections 400 μm in thickness, the tumor was washed with cold phosphate-buffered saline (PBS), then fixed for 10 min in 2% paraformaldehyde solution, washed with PBS, and embedded in 2% agarose. Then, the preparation was cut into 400-µm layers, and the sections were additionally fixed with a 2% paraformaldehyde solution for 5 min. After that, immunostaining was performed with fluorescently labeled Abs against HER2, CD45, Ki-67, CD31, PD-L1, and reticular fibers. The penetration of the Abs into the 400-μm-thick section and its complete staining throughout the section took a total of 18 h. In order to achieve high optical transparency, the sections were sequentially incubated for 1 h in 20, 50, and 80% D-fructose solutions at 25 °C under gentle stirring. The prepared sections were scanned with a confocal microscope to form a full 3D model of the tumor and its microenvironment from several successive sections (Figure 4). This 3D analysis revealed colocalization of different markers. For example, HER2^+^, PD-L1^+^ cells were concentrated at the tumor periphery, while HER2^+^, PD-L1^+^ cells were closer to the center. The highest expression of PD-L1 was observed at the tumor periphery, because apparently, not only HER2-positive cancer cells, but also stromal components expressed it. Clusters of CD45^+^ cells corresponding to tumor-infiltrating lymphocytes were also localized at the tumor margins, which confirmed that the PD-L1 signaling pathway prevents immune cells from entering the tumor.

This process of bleaching the sample is necessary to overcome certain limitations of 3D imaging, i.e., the high radiation absorption and high background signal levels due to the presence of lipids and other components that can cause optical signal absorption and autofluorescence in the visible spectrum. Below, we will discuss how different types of fluorescent tags can be used to address this limitation. The second common limitation is the photobleaching of fluorescent labels and reducing their fluorescence levels, which occurs during linear scanning with a confocal microscope. Park et al. [97] suggested dealing with this problem by using the Point Accumulation for Imaging in Nanoscale Topography (DNA-PAINT) method [98] for 3D fluorescence imaging of thick tissue sections. Target-specific Abs conjugated to oligonucleotides 10 to 30 nucleotide residues in length and complementary oligonucleotides conjugated to organic fluorescent dyes served as the imaging labels. Frozen tissue samples fixed in sucrose were cut into 100-μm-thick blocks and stained with the detection antibodies. The blocks were enclosed between the glass slides, with space left for the imaging buffer solution, containing conjugates of oligonucleotides and organic fluorescent dyes, to flow through. The samples were then scanned with a confocal microscope in this flow cell, in which the imaging buffer was replaced every hour using a syringe pump. The fluorescent oligonucleotide labels contained in the solution were not spatially fixed; therefore, they were constantly moving, and the signal was not accumulated at a specific point. In contrast, when a fluorescent label was bound to the complementary oligonucleotide, it became fixed in space, so that the fluorescent signal accumulated and was detected. The interaction properties of the oligonucleotides are chosen so that this method makes it possible to replace photobleached fluorescent labels with fresh ones in the process of scanning different layers and, hence, ensure highly sensitive detection.

Various techniques are available to study tissue slices, which differ in maximum visualization depth, resolution of the obtained image, and possibility of quantitative and multiplex analysis. Modern and advanced methods of analyzing histological structures and comparison of their benefits and limitations presented in Table 2. In spite of development of new methods to study histological structure of tissues, it should be noted that with increase of maximum detection depth, the spatial resolution of the obtained image decreases, which negatively affects the result. The mentioned methods are suitable only for in vitro studies, while for clinical diagnostics, safer and noninvasive methods are used, which allow to study objects at a greater depth, although with a lower spatial resolution. In the last several years, deep learning and neural networks have been actively implemented in histopathology images of BC, which help in its detection, segmentation, and classification [99,100].

These examples suggest the basic requirements for ideal fluorescent labels: they should brightly fluoresce in the infrared or near-infrared spectral region in order to fall within the transparency window of biological tissues, have a high photostability to allow prolonged layer-by-layer scanning with a confocal microscope without the loss of the fluorescent signal, and have a narrow fluorescence spectrum and a wide range of possible colors to be used in multiplexed detection. Fluorescent nanocrystals (NCs), which have been increasingly used for biological imaging in the past decade, possess all these properties. They will be discussed in detail below.

## 5. Fluorescent Labels for Multiplexed 3D Imaging

**Figure 5:**
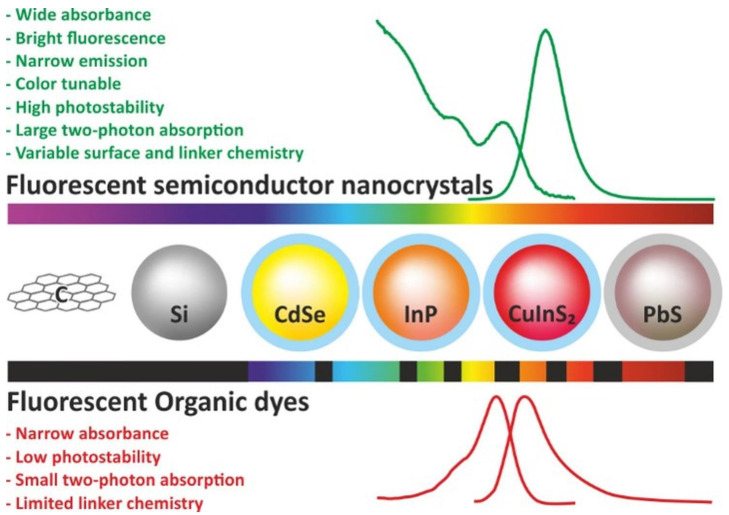
Comparison of the main properties of fluorescent nanocrystals and organic fluorescent dyes.

Although the sensitivity of fluorescence detection is lower compared with methods based on ionizing radiation detection, such as positron-emission tomography (PET) and single-photon emission computed tomography (SPECT), fluorescence detection has been widely used in biological research for several decades. Moreover, its use has not only improved the existing techniques, but also offered new opportunities, including multiplexed IHC assay [114], 3D imaging of biological tissues [115], in vivo imaging [116], study of interactions between biomolecules [117], and many other approaches. This is due to the considerably lower costs of both the detection equipment and the fluorescent labels themselves. In addition, the labels for PET and SPECT are radioactive, which makes their use less safe. The development of fluorescence 3D imaging methods was boosted by the appearance of organic dyes that fluoresce in the infrared and near-infrared spectral regions, which allowed signal detection in the optical transparency range of biological tissues, thus increasing the ratio of the signal to the autofluorescence noise of biological tissues and increasing the detection depth and spatial resolution [118,119]. Fluorescent NCs are increasingly more popular compared to organic dyes due to their high brightness, photostability, broad excitation spectra with narrow fluorescence peaks, and other optical and physical properties, such as hydrophilicity, which give them advantage over organic fluorescent dyes (Figure 5) [120,121,122]. Nevertheless, many fluorescent NCs are toxic, which is related to either their chemical composition or their physical properties [123]. The NC physical properties, including the NC shape, size, and surface charge, can be modified to make them less toxic, but the toxicity determined by the chemical composition can only be reduced by either coating NCs with a stable shell or designing new NCs consisting of nontoxic elements. The NC toxicity is a problem not only for in vivo imaging, but also for in vitro studies, because NCs can enter the body in a natural way, e.g., by inhalation or through the skin. A fluorescent NC consists of either a bare core alone or a core coated with an epitaxial inorganic shell. The core consists of elements of groups III–V, II–VI, or IV–VI of the periodic system, carbon, silicon, or graphene. The shell serves not only for reducing toxicity, but also for increasing the NC resistance to environmental factors and improving their optical properties [124]. NCs with cores of heavy metal compounds, such as CdSe, CdTe, and PbS, were initially the most widespread due to their easy synthesis and unique optical properties [125,126]. However, the current trend is to use the nontoxic cadmium-free and lead-free fluorescent NCs made of silicon [127], carbon [128], graphene [129], ZnS [130], CuInS_2_ [131], AgInS_2_ [132], or InP [133].

The prerequisites for highly sensitive multiplexed 3D detection are a high quantum yield (QY) of fluorescence for providing a sufficient level of useful signal; relatively narrow, nonoverlapping fluorescence spectra for effectively distinguishing the signals from different NCs detected simultaneously; and the possibility to excite fluorescence in the infrared region or in the two-photon mode for minimizing the background autofluorescence of tissue samples and ensuring deeper penetration of the radiation. Wang et al. [130] showed that NCs with a ZnS core have a wide fluorescence spectrum (360–600 nm) with a maximum at 408 nm, which makes them unsuitable for multiplexed detection. NCs with a CuInS_2_ core have a large two-photon absorption cross section [134], but a relatively low fluorescence QY and, similar to ZnS NCs, a wide fluorescence spectrum; hence, they are not the best choice for multiplexed detection either. The fluorescence spectrum of NCs is determined by their size and composition [135], but some materials are poorly suitable for making NCs of a desirable size or separating NCs of different sizes. For example, Raevskaya et al. [136] have reported that the synthesis of water-soluble NCs with an AgInS_2_ core, which have a relatively narrow fluorescence spectrum, involves a laborious procedure of size-selective precipitation for obtaining NCs of different sizes. NCs with an InP core, whose fluorescence wavelength is closely correlated with the core size [137], are a better nontoxic alternative to CdSe-based NCs [138]. The only problem with their synthesis that existed until recently was the need to use highly toxic and explosive phosphorus precursors, such as tris(trimethylsilyl)phosphine. However, there are new techniques for the synthesis of InP NCs from less dangerous precursors, i.e., white phosphorus [139], small InP clusters [140], and others [141]. Originally, InP NCs had a fluorescence QY as low as 5% or less in aqueous solution and, in addition, quickly degraded after synthesis because their surface was rapidly oxidized. To overcome this problem, Clarke et al. [142] suggested capping the InP core with a shell made of ZnS because this material has a similar crystal lattice. This allowed them to obtain core/shell NCs with a fluorescence QY of 85% in organic solvents and 57% in aqueous solutions [141]. Kim et al. [143] obtained an even higher fluorescence QY (95%) by applying a ZnSe/ZnS multilayer shell over the InP core.

To detect the signals from individual fluorophores, which is often required for biological imaging, the labels should be as little susceptible to photobleaching (a decrease in fluorescence intensity with time) as possible and exhibit the minimal possible blinking, i.e., they should remain in the emitting state as long as possible. NCs are much more resistant to photobleaching than organic dyes, but blinking remains an issue. Reid et al. [144] described the synthesis of InP/ZnS NCs with a thick shell that allowed NCs to emit for more than 95% of the time; however, this shell increased the NC size, which was not ideal for biological detection. Another approach to obtaining nonblinking, nontoxic InP-based NCs is to apply a multilayer inorganic shell, e.g., a ZnSe/ZnS shell, and then treat the surface with hydrofluoric acid to reduce the surface nonradiative recombination [145]. This approach yields nonblinking NCs with a fluorescence QY of about 90% that have almost no effect on cell viability and provides a resolution 2.6 times better than that of wide-field microscopy.

Furthermore, fluorescent NCs have a wide absorption spectrum, which allows NCs of different colors to be excited with a single light source. This not only requires a simpler setup for fluorescence imaging, but also accelerates the procedure. Fluorescent NCs also have two other features that make it possible to considerably increase the sensitivity of imaging by reducing the background signal. Specifically, organic fluorescent dyes have a fluorescence lifetime of about 1–10 ns, whereas the NC fluorescence lifetime is about 10–100 ns [146]. The lifetime of the autofluorescence of biological molecules is also on the order of 1–10 ns, e.g., about 7.5 ns for phenylalanine [147] and about 4 ns for flavin mononucleotide [148]. Endogenous biological molecules also fluoresce when the signal from organic fluorescent dyes is detected, thus generating a strong background noise. The fluorescent signal from NCs can be detected for several tens of nanoseconds after the autofluorescence signal has already decreased. This advantage is already widely used in Fluorescence Lifetime Imaging Microscopy (FLIM) [149,150]. In addition, fluorescent NCs have a larger two-photon absorption cross section than organic fluorescent dyes, averaging from 2 × 10^–47^ to 4.7 × 10^–46^ cm^4^ s photon^–1^ versus 1 × 10^–49^ cm^4^ s photon^–1^ [146]. This allows NC fluorescence to be excited in the infrared range, thereby reducing both the absorption of radiation by the molecules of the sample and the excitation of autofluorescence, which further increases the contrast and sensitivity of imaging [151,152].

Below, we will consider how 3D imaging probes can be further improved by using ultrasmall biological capture molecules.

## 6. Capture Molecules for 3D Imaging

**Figure 6:**
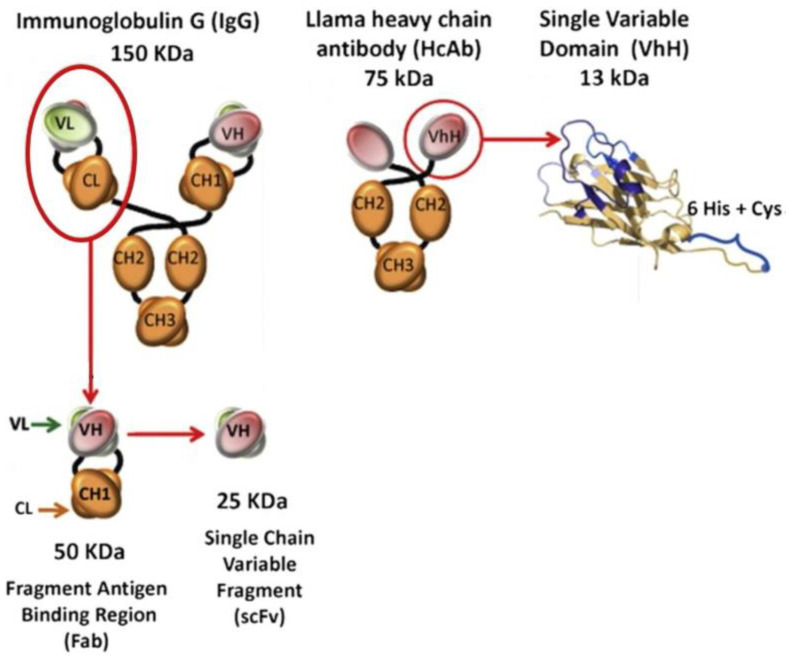
Structure of different types of antibodies and their functional fragments and domains. Adapted from Sukhanova et al. [157] Copyright (2023), with permission from Elsevier.

High sensitivity and specificity of any system for detection of biological analytes rely on recognition molecules. Nature has created a mechanism for the synthesis of ideal recognition molecules, Abs, and modern science and biotechnology have made it possible to obtain Abs against specific analytes and in practically unlimited amounts by using, e.g., hybridoma technologies [153]. In addition, highly sensitive and specific Abs have been obtained by bioengineering methods, including directed evolution of the antigen-binding sites [154]. In addition to the most common Abs, immunoglobulins G (IgG), there are other, noncanonical Abs, which are also successfully used for detecting biological molecules [155]. The problem with IgG is their large molecular weight (about 150 kDa) and, hence, size, which complicates their movement in tissues due to a significant concentration gradient, causes nonspecific staining, etc. The antigen-binding fragment of IgG consists of the variable domain of the heavy chain (VH) and the variable domain of the light chain (VL) (Figure 6). They can be separated from the rest of the Ab molecule and joined, through a linker, into the so-called single-chain variable fragment (scFv) with a molecular weight of about 25 kDa. Owing to their small size, scFvs are better suited for biological imaging. For example, Fan et al. [156] have described the use of scFvs for the detection of CD25-positive tumors. However, this size is not the lower limit for Ab-derived capture molecules. For example, camelids, such as llamas, produce noncanonical Abs that consist of only the heavy chain and have no light chain at all. Their variable fragment, which serves for the specific binding of antigens, is represented by only one VH domain. Because of their small size and structure, they are termed nanobodies or single-domain antibodies (sdAbs).

**Figure 7:**
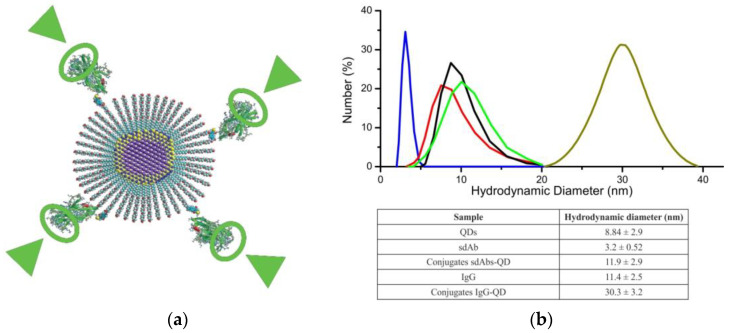
The structure and size of oriented conjugates of single-domain antibodies (sdAbs) and fluorescent nanocrystals (NCs) to be used as diagnostic probes. (**a**) The NC is conjugated with sdAbs via a single Cys residue specifically integrated into the sdAb C-terminus. The antigen-binding domain of each sdAb (green circles) is exposed to the outside and remains functionally active (green arrows). (**b**) Comparative dynamic light scattering measurements of sdAbs (shown in blue), NCs (shown in red), monoclonal antibody (IgG) (shown in black), sdAb–NC conjugates (shown in light green), and monoclonal antibody–NC conjugates (shown in ocher). Adapted from Sukhanova et al. [157] Copyright (2023), with permission from Elsevier.

SdAbs are ten times smaller than human IgG, have a molecular weight of about 12–15 kDa, consist, on average, of slightly more than 100 amino acid residues, have an elongated shape, and are about 2 nm in length. Their compactness gives sdAbs a number of advantages; in particular, they are more resistant to environmental factors, including high temperatures and detergents, and hence, have a high conformational stability, which is important for specific and sensitive antigen binding [158]. In addition, their smaller size helps them to penetrate deeper into tissues and bind target epitopes that are inaccessible for full-size Abs [159]. An important property of sdAbs is their high hydrophilicity allowing them to remain stable and active in aqueous solutions, which is necessary for immunological detection methods [160]. The method of conjugation of biological capture molecules with fluorescent labels is also worth considering, because it may substantially affect the stability and aggregation properties, as well as preservation of the function of antigen-binding sites. If the conjugation is nonoriented, it can occur at any site and any amino acid residue of the Ab molecule, but covalent binding through the primary amine of lysine residues is most commonly used, because they are positively charged and many of them are sufficiently exposed into the solution [161,162]. However, since lysine is one of the most abundant amino acids in the antibody structure, conjugation through it leads to conformational changes in Ab molecules, accompanied by complete or partial degradation of their functional activities. The cysteine side chain is also used for oriented conjugation, but the thiol groups of a cysteine residue in a native protein rarely occur in the free reduced form, usually forming disulfide bonds with one another. Traditionally, sdAbs are obtained by expressing the corresponding gene constructs in microbial cells. This biotechnological route of synthesis yields modified genetically engineered constructs that can be conjugated with fluorescent NCs in an oriented manner. For example, Sukhanova et al. [157] obtained highly oriented conjugates of sdAbs and fluorescent NCs by binding a linker with a C-terminal cysteine residue to the sdAb molecule for linking the sdAbs to the NC surface functionalized with polyethylene glycol derivatives. This procedure was used to fabricate fluorescent probes with a hydrodynamic diameter of about 12 nm, which is comparable in size with full-length IgG (Figure 7). There are already a number of examples of the production and successful use of sdAbs against EGFR [163]; HER2 [164]; immune response checkpoint markers, including PD-L1 [165], LAG-3/CD233 [166] CD206/MMR [167], and VSIG4 [83]; and many other markers relevant to BC diagnosis and treatment.

Toxicity of nanoprobes composed of fluorescent nanocrystals and sdAbs is mainly determined by nanocrystals toxicity that are discussed previously. When talking about the side effects of sdAbs, the more important is to mention its immunogenicity with their potential to cause unwanted immune reactions and not just talking about their toxicity. For example, Cortez-Retamozo et al. studied cancer cell labeling using sdAbs in mouse models [168]. They intravenously injected 10 µg of sdAbs daily for three days, after which they examined blood samples and found no traces of antibodies against the injected sdAbs. Similarly, their in vitro studies on lymphocytes showed no significant increase in IFN-γ or IL-10 synthesis. Coppieters et al. also showed that sdAbs did not cause an immune response in mice [169]. Kibria at al. investigated immunogenicity of sdAbs in Jcl:ICR mice [170]. They tested two heat-aggregated anti-EGFR sdAbs derived from *E. coli* and detect immunogenicity only for unfolded and aggregated sdAbs. The minimum immunogenicity for sdAbs produced in mammalian cell cultures, because of similar posttranslational modifications and folding processes [171]. In addition, to reduce the risk of immunogenic effects, a so-called humanization procedure is used, in which part of the sequence of amino acid residues of sdAbs is replaced with the corresponding human [172]. To date, at least three dozen clinical trials of drugs that are based on sdAbs have been completed. Most of the trials did not show immunogenicity of sdAbs or their immunogenicity did not exceed that of fully human or humanized IgGs [173]. Taking into account the above, sdAbs and fluorescent NC based nonoprobes are nontoxic for histological examinations of tissue sample in vitro. The low immunogenicity of adAbs makes it possible to create not only highly specific drugs based on them but also create vesicles for target delivery of pharmaceutical compounds and diagnostics. There are many examples of using sdAbs as targeting agents in vivo for cancer cell and metastasis detection by optical methods [174] or by SPECT/CT [175,176]. Recently, Feng at al. investigated ^131^I-labeled anti-HER2 sdAb for the radiopharmaceutical therapy of HER2-expressing BC in mice [177]. Due to the small size of the ^131^I-labeled sdAb, they spread quickly in the body, and the maximum concentration in the tumor was observed after one hour. After only 16 h, the concentration of ^131^I-labeled sdAb in various organs fell below the threshold of detection and error, while in the tumor, the concentration of conjugates dropped only three-fold a day after injection. The small size of recognition molecule conjugates with NCs allows them to penetrate the blood–brain barrier, which makes them relevant for the detection and treatment of brain diseases too [178]. At the moment, theranostics based on sdAbs and NC conjugates are being actively developed, although their future clinical application is questionable without solving questions about NC potential toxicity. Currently, there are many companies which develop and sell sdAbs, including R&D systems, Abnova, Creative Biolabs, Genscript, etc., as well as companies which produce and sell fluorescent NC, for example, Nanografi Nano Technology, Ocean NanoTech, Thermo Fisher Scientific, and others. The simple conjugation procedure makes them a solid alternative to existing commercial fluorescently and enzymatically labeled antibodies for immunohistochemical tissue investigations.

## 7. Conclusions

Today, IHC as a method for examining tumor tissue is the most common not only in clinical laboratories, but also in scientific research. Although the method has become routine, laboratories worldwide use different protocols for permeabilization, fixation, and staining, as well as different Abs for detection of target biomarkers; therefore, the results vary between laboratories. The increased accuracy of studies and the possibility of analyzing laboratory results worldwide and comparing them with the actual clinical picture of BC over a long period of time has made it possible to revise and update the methodological guidelines for the assessment of IHC data on an annual basis. At the same time, only four markers, ER, PR, Ki-67, and HER2, are recommended for clinical use and classification of tumor subtypes [179], although, as noted above, research laboratories and some clinical diagnostic laboratories already use considerably more biomarkers. Comprehensive analysis of cancer markers is also required for selecting the most efficacious hormonal therapy, which is increasingly being used for the treatment of certain types of BC. All this calls for a new, advanced method of analyzing the histological structure of tumor tissues and tumor microenvironment that would provide systemic information on multiple biomarkers, which is necessary for more effective treatment of BC based on a personalized approach.

Malignant tumors are highly heterogeneous, with only part of the cells expressing a given marker detectable, e.g., using IHC methods. Heterogeneity concerns variations of the organization of cells, characteristics of the extracellular matrix, and structure of tumor infiltration by immune cells. These variations cannot be assessed without systematic detailed histological examination. The high heterogeneity of the tumor makes it impossible to assess the complex topography using individual thin sections usually analyzed by IHC methods; hence, there is a need for the examination of thicker specimens. There are approaches where several consecutive sections are examined by traditional IHC methods, with staining for many biomarkers, but this is a technically complex and time-consuming procedure.

The main problem with staining thick sections is that radiation in the visible range is effectively absorbed by biological molecules, which attenuates the useful signal and enhances the background signal [180]. Several advanced 3D imaging approaches circumvent these limitations. The first approach employs two-photon excitation, where the fluorophore is excited upon absorption of two photons with a longer wavelength, such as near-infrared photons. Radiation in this spectral range is less absorbed and scattered by biological tissues, causing a weaker autofluorescence signal [151]. The second approach is based on confocal microscopy based on the ability to filter optical radiation coming from outside the focal plane of the objective lens, which allows obtaining a series of images located at different depths of the sample [103]. At the same time, to visualize thick tissue sections, an optical tissue cleaning procedure can be applied, which includes removal of pigments and lipids, but these procedures are technically laborious and long, and in the process of sample preparation, important biomarkers can be lost and the structure of the extracellular matrix can be disturbed [181].

Nanocrystals have a number of advantages as fluorescent labels for 3D imaging:

- Narrow fluorescence spectra in a wide range of wavelengths, making fluorescent NCs suitable for multiplexed detection;
- A high photostability allowing long-term scanning and signal accumulation and simplifying tissue staining procedures;
- The possibility to tune the fluorescence spectrum by varying the NC size and composition, including the possibility to obtain NCs emitting in the infrared and near-infrared spectral ranges;
- A large two-photon absorption cross section allowing excitation in the infrared range, thus ensuring deeper penetration of radiation, a stronger useful signal, and a weaker background signal;
- Low blinking allowing detection of signals from individual fluorophores;
- A long fluorescence lifetime providing conditions for FLIM.

Single-domain antibodies and probes based on them also have a number of unique properties that make them advantageous as 3D imaging tools:

- A small size allowing a greater number of capture molecules to be linked to the fluorescent label;
- A small size of sdAb–NC conjugates promoting tissue penetration and detection of hidden epitopes inaccessible for full-length antibodies;
- The possibility of obtaining a functionally active conjugate with the highest possible avidity where all sdAb molecules are bound to the NC in a strictly oriented manner;
- High stability and hydrophilicity allowing the staining and signal detection within a wider range of physical and chemical parameters, thus optimizing and simplifying the permeabilization, fixation, and staining procedures.

As shown in this review, only integrated detection of multiple biomarkers allows the BC type to be accurately identified, which is essential for correct prognosis and selection of effective immunotherapy and for development of new approaches to BC treatment. Three-dimensional optical imaging is a relatively low-cost and highly informative method for examining samples of tumor tissue and tumor microenvironment. This approach can be made even more effective by using imaging probes consisting of sdAbs and fluorescent NCs, offering a number of advantages over more routine optical 3D imaging tools.

## Figures and Tables

**Table 1:** Expression of myoepithelial markers in tumors of different types. Adapted from Ref. [13].

Proportion of Epithelial Cells Expressing Ki-67 *	Normal Tissue	Hyperplasia	Mammary Intraepithelial Neoplasia	Carcinoma
	7%	39%	55%	72%
SMA	++	++−	+−−	-
SMMHC	++	++	++−	-
CK14	++	++	++−	-
p63	++	++−	+−−	-

* Proportion of cells expressing the marker: ++, >75%; ++−, <75%; +−−, <25%; -, none.

**Table 2:** Comparison of different methods of analyzing histological structures.

Imaging Method	Multiplexing	Quantity	Depth of Imaging	Resolution	Refs.
Phase-contrast microscopy	few	semi	~1–10 μm	~10 nm	[101,102]
Confocal microscopy	yes	yes	~100–100 μm	~100 nm	[103,104]
Multiphoton microscopy	yes	yes	~100–1000 μm	~10–100 nm	[105,106]
Optical coherence tomography	no	semi	~1 mm	~1–10 μm	[107,108]
Raman spectroscopy	few	semi	~1–10 mm	~10–100 nm	[109,110]
Fluorescence molecular tomography	yes	yes	~1 cm	~1 mm	[111]
Photoacoustic microscopy	few	yes	~1–10 cm	~10–100 μm	[112,113]

## Data Availability

Not applicable.
